# The Current Role of Adjuvant Chemotherapy in Locally Advanced Nasopharyngeal Carcinoma

**DOI:** 10.3389/fonc.2020.585046

**Published:** 2021-02-24

**Authors:** Lin Su, Lei She, Liangfang Shen

**Affiliations:** ^1^ Department of Oncology, Xiangya Hospital, Central South University, Changsha, China; ^2^ Department of Clinical Pharmacology, Xiangya Hospital, Central South University, Changsha, China; ^3^ Institute of Clinical Pharmacology, Central South University, Hunan Key Laboratory of Pharmacogenetics, Changsha, China

**Keywords:** locally advanced nasopharyngeal carcinoma, adjuvant chemotherapy, radiotherapy, survival, toxicity

## Abstract

Nasopharyngeal carcinoma (NPC) is one of the most common malignant tumors of the head and neck, and it originates from the mucous epithelium of the nasopharynx. Because it is “hidden”, the symptoms of NPC can easily be missed, and more than 70% of patients present with locally advanced disease at diagnosis. Concurrent radiation therapy with chemotherapy can significantly improve regional control of NPC. At present, distant metastasis is the main cause of treatment failure. At the end of the 20th century, clinical trial No. IG0099 in the United States confirmed the effectiveness of adjuvant chemotherapy (AC) for the first time. However, in the past 20 years, various clinical trials and meta-analyses conducted globally have yielded contradictory results regarding the effect of AC on locally advanced NPC. AC has changed from category 1 to the current category 2A in the National Comprehensive Cancer Network (NCCN) guidelines, and it remains controversial whether AC can significantly improve the survival of NPC patients. Here, we comprehensively analyzed the role of AC in locally advanced NPC by comparing some treatment methods. We conclude the role of AC in treating locally advanced NPC, based on the studies presented, remains undefined but is associated with increased toxicity.

## Introduction

Nasopharyngeal carcinoma (NPC) is one of the most commonly diagnosed malignant tumors of the head and neck, and it originates from the mucous epithelium of the nasopharynx. NPC accounts for approximately 60% of all head and neck tumors (1). According to the International Cancer Research Agency, there were 129,079 new cases of NPC worldwide in 2018, and the age-standardized incidence rate of the population was approximately 1.5/100,000, accounting for 0.7% of the total number of new cancers globally (2, 3). The incidence of NPC has obvious regional, ethnic, and familial epidemiological characteristics. The incidence of NPC varies widely from region to region and is prevalent in East Asia, Southeast Asia, East Africa, and North Africa. China accounts for approximately 47.7% of all new cases of NPC globally (4). Nonetheless, China presents significant regional differences in the incidence of NPC, with the highest prevalence being found in South China, including Hunan, Guangdong, Guangxi, and Fujian (5). The main treatment for NPC is radiotherapy (RT) because of its hidden anatomic location, proximity to important blood vessels and nerves, and high sensitivity to this treatment. Clinically, radical RT is commonly administered for early NPC as it provides the best results. Due to the special location and occult nature of NPC, more than 70% of patients present with locally advanced stage at diagnosis (6). At present, the local control (LC) rate for locally advanced NPC is more than 90%. The efficacy of concurrent chemoradiotherapy (CCRT) is widely recognized; however, distant metastasis is the main cause of treatment failure (7–10). The first edition of the National Comprehensive Cancer Network (NCCN) guidelines in 2020 classified CCRT plus adjuvant chemotherapy (AC) as category 2A, and CCRT alone as category 2B. However, the efficacy of AC is still controversial. Here, we review the literature and the research progress regarding the use of AC for the treatment of locally advanced NPC in order to provide a point of reference for more accurate treatment of this disease.

## Concurrent Chemoradiotherapy Plus Adjuvant Chemotherapy Versus Radiotherapy Alone

RT remains the most important treatment for NPC; however, RT alone is not enough to treat locally advanced NPC (**Table 1**). At the end of the 20th century, a phase III, randomized, controlled clinical trial (No. IG0099) in the United States showed for the first time that, compared with RT alone, CCRT+AC could significantly benefit patients with locally advanced NPC. The 3-year overall survival (OS) rate increased by 31%. This trial included 193 patients with NPC who were diagnosed as stage III and IV. Among them, 147 cases could be evaluated. All the patients received conventional RT. Among them, 78 received a three-week regimen of 100 mg/m^2^ of concurrent cisplatin chemotherapy; after CCRT, the patients received three cycles of AC (80 mg/m^2^ cisplatin and 1000 mg/m^2^ fluorouracil every four weeks). The results showed that the three-year OS rate of patients in the CCRT+AC group and RT group was 78% and 47% (*P*<0.005), while the three-year progression-free survival (PFS) rate was 69% and 24% (*P*<0.001), respectively, indicating that the patients who received CCRT+AC had greater treatment benefits (11). Furthermore, in a secondary analysis, Al-Sarraf et al. included patients who lacked preregistration documentation and previously analyzed patients. In this larger data set, 93 in the combined group and 92 in the RT group. The five-year OS rate of the two groups was 67% and 37% (*P*<0.01), while the five-year PFS rate of the two groups was 58% and 29%, respectively (*P*<0.01) (12). As a consequence, the “three cycles of CCRT (cisplatin) plus three cycles of AC (PF regimen)” treatment regimen became the standard regimen for the treatment of locally advanced NPC in North America. Since then, NCCN guidelines have recommended CCRT+AC as a category 1 treatment for locally advanced NPC. Nonetheless, this trial still had some limitations. First, the incidence of NPC is not high in North America. Second, the main pathological types of NPC in the study were poorly differentiated and undifferentiated carcinoma, but 55% of the patients presented with well-differentiated squamous cell carcinoma. Finally, only 63% and 55% of the patients completed three cycles of concurrent chemotherapy (CCT) and three cycles of AC, respectively.

**Table 1:** Concurrent chemoradiotherapy plus adjuvant chemotherapy versus radiotherapy alone.

References	Inclusion period	Comparison	Number of patients		Stage	Concurrent chemotherapy	Survival endpoints	With AC	No AC	*P*
Al-Sarraf et al. (11)	1989–1995	CCRT+AC/RT alone	78/69		III–IV (M_0_), AJCC	cisplatin	3-year OS	78.00%	47.00%	0.005
							3-year PFS	69.00%	24.00%	<0.001
Al-Sarraf et al. (12)	1989–1995	CCRT+AC/RT alone	93/92		III–IV (M_0_), AJCC	cisplatin	5-year OS	67%	37%	<0.01
							5-year PFS	58%	29%	<0.01
Wee et al. (13)	1997–2003	CCRT+AC/RT alone	111/110		T_3-4_N_x_M_0_ or T_x_N_2-3_M_0_,AJCC/UICC (1997)	cisplatin	3-year OS	80.00%	65.00%	0.0061
							3-year DFS	72.00%	53.00%	0.0093
Lee et al. (14)	1999–2004	CCRT+AC/RT alone	172/176		T_1-4_N_2-3_M_0_, 5th AJCC/UICC	cisplatin	3-year OS	78.00%	78.00%	0.97
							3-year FFS	72.00%	62.00%	0.027
Lee et al. (15)	1999–2004	CCRT+AC/RT alone	172/176		T_1-4_N_2-3_M_0_, 5th AJCC/UICC	cisplatin	10-year OS	62%	49%	0.047
							10-year FFS	62%	50%	0.010
Ng et al. (16)	1999–2004	CCRT+AC/RT alone	223/218		III–IVB, 5th AJCC/UICC	cisplatin	10-year OS	60%	50%	0.044
							10-year FFS	62%	52%	0.016
Chen et al. (17)	2002–2005	CCRT+AC/RT alone	158/158		III–IVB, 5th AJCC/UICC	cisplatin	2-year OS	89.9%	79.7%	0.003
							2-year FFS	84.6%	72.5%	0.001
Chen et al. (18)	2002–2005	CCRT+AC/RT alone	158/158		III–IVB,5th AJCC/UICC	cisplatin	5-year OS	72%	62%	0.043
							5-year FFS	72%	62%	0.020
Lin et al. (19)	1993–1999	CCRT/RT alone	141/143		III–IV (M_0_), AJCC(1992)	cisplatin, fluorouracil	5-year OS	72.3%	54.2%	0.0022
							5-year PFS	71.6%	53.0%	0.0012
Chan et al. (20)	1994–1999	CCRT/RT alone	174/176		II–IV (M_0_), AJCC (1997)	cisplatin	5-year OS	70.3%	58.6%	0.065
							5-year PFS	60.20%	52.10%	0.16

AC, adjuvant chemotherapy; RT, radiotherapy; CCRT+AC, concurrent chemoradiotherapy plus adjuvant chemotherapy; OS, overall survival; PFS, progression-free survival; FFS, failure-free survival; DFS, disease-free survival; T, tumor; N, node; M, metastasis; CCRT, concurrent chemoradiotherapy; AJCC/UICC, American Joint Committee on Cancer and Union for International Cancer Control.

East and Southeast Asia have a high incidence of NPC, and 90% of the pathological types are poorly differentiated and undifferentiated carcinoma. Consequently, several trials have explored the efficacy and adverse reactions of this regimen in areas with a high incidence of NPC. In Singapore, Wee et al. conducted a prospective randomized controlled clinical trial of this regimen. They enrolled 221 patients (T_3–4_N_x_M_0_ or T_x_N_2–3_M_0_, American Joint Committee on Cancer and Union for International Cancer Control [AJCC/UICC] fifth edition staging) with WHO type II or III histology. A total of 110 patients were assigned to the RT treatment group and 111 to the CCRT+AC treatment group. Of these, 71% completed three cycles of CCT, and 57% completed 3 cycles of AC. Statistical analysis showed that after a median follow-up of 3.2 years, distant metastasis occurred in 38 cases in the RT group and 18 cases in the CCRT+AC group. The two-year cumulative incidence rate was 17% (95% CI=14–20; *P*=0.0029). The hazard ratio (HR) for disease-free survival (DFS) was 0.57 (95% CI= 0.38–0.87; *P*=0.0093). The two-year OS rate of the RT group and the CCRT+AC group was 78% and 85%, while the three-year OS rate was 65% and 80%, respectively; the HR of the OS rate was 0.51 (95% CI=0.31–0.81; *P*=0.0061). The results of this study showed that CCRT+AC could significantly improve DFS, distant metastasis-free survival (DMFS), and OS compared with RT alone (13).

Lee et al. also conducted a prospective, randomized, controlled clinical trial of this regimen in Hong Kong. This trial enrolled 348 patients (T_1–4_N_2–3_M_0_, AJCC/UICC fifth edition staging, with nonkeratinizing carcinoma histological features). Of these, 176 were assigned to the RT treatment group and 172 to the CCRT+AC treatment group. In total, 65% of the patients completed all six cycles of chemotherapy and 79% completed more than five cycles. Statistical analysis of the results showed that, after a median follow-up of 2.3 years, the three-year DFS rate of the patients in the CCRT+AC group was significantly better than that of the RT treatment group (72% *vs.* 62%, *P*=0.027), mainly due to an improved local–regional control rate (92% *vs.* 82%, *P*=0.005). However, the three-year DMFS rate did not show a significant improvement (76% *vs.* 73%, *P*=0.47), and the three-year OS rate was similar between the two groups (78% *vs.* 78%, *P*=0.97). In addition, the acute toxicity associated with the CCRT+AC treatment was significantly higher than that associated with RT treatment (84% *vs.* 53%, *P*=0.001), and the late toxicity related to this treatment was also higher (three years later, 28% *vs.* 13%, *P*=0.024) (14). Significant improvements in tumor control were maintained after 10.7 years follow-up; 10-year survival analysis showed that patients in the CCRT+AC group displayed a better LC rate (87% *vs.* 74%; *P*=0.003), failure-free survival rate (FFR) (62% *vs.* 50%; *P*=0.01), and PFS rate (56% *vs.* 42%; *P*=0.006) compared with patients treated with RT alone. Surprisingly, the 10-year OS rate of the CCRT+AC group showed a statistically significant improvement (62% *vs.* 49%; *P*=0.047). However, the effect on the DMFS rate was still not significant (68% *vs.* 65%; *P*=0.24). With longer follow-up time, the differences in toxicity and side effects between the two groups gradually decreased. The late toxicity at 10 years was 52% *vs.* 47% (*P*=0.20), respectively, and 4.1% and 2.8% of the patients, respectively, died due to treatment-related toxic reactions; the incidental/unexplained mortality rate was 15.1% and 13.1%, respectively (15).

In the latest combined analysis of two randomized studies (NPC-9901 and NPC-9902 trials), a total of 441 patients (III–IVB, AJCC/UICC fifth edition staging, nonkeratinizing carcinoma pathological type) were assigned to a RT treatment group (*n*=218) and a CCRT+AC treatment group (*n*=223). Ten-year survival analysis showed that patients in the CCRT+AC group had significantly better FFR (62% *vs.* 52%, *P*=0.016), PFS rate (56% *vs.* 44%, *P*=0.008), and OS rate (60% *vs.* 50%, *P*=0.044). Exploratory studies had shown that two or three cycles of CCT could not improve the disease control (DC) rate. Only patients who continued to receive two or more cycles of AC (cisplatin–fluorouracil) achieved significant improvements in the DMFS rate (73% *vs.* 65%, *P*=0.037) and achieved the greatest survival benefits (16).

Similarly, 316 patients (III–IVB, AJCC/UICC fifth edition staging, nonkeratinizing carcinoma pathological type) from the Cancer Prevention and Treatment Center of Sun Yat-sen University in China were included in a prospective phase III clinical study. Of the 316 patients, 158 were assigned to the CCRT+AC group and 158 to the RT treatment group. Slightly different from the IG0099 clinical trial, a weekly CCT regimen was adopted in this trial (cisplatin 40 mg/m^2^, d1, once a week, seven times in a row). Both groups were treated with radical conventional fractionation RT. In the CCRT+AC group, 91.1% of the patients completed more than five cycles of CCT, 84.2% completed more than six cycles of CCT, 68.4% completed seven cycles of CCT, and 61.4% completed three cycles of AC. The proportion of acute side effects of grade 3 or higher in the CCRT+AC group and the RT group was 62.6% and 32.3%, respectively (*P*=0.000). The two-year local recurrence-free survival (LRFS) rate, DMFS rate, DFS rate, and OS rate of these two groups was 98.0% *vs.* 91.9%, 86.5% *vs.* 78.7%, 84.6% *vs.* 72.5%, and 89.8% *vs.* 79.7%, respectively (17). Long-term observation showed that, after a median follow-up of 70 months, the five-year OS rate was 72% in the CCRT+AC group and 62% in the RT group (HR=0.69; 95% CI=0.48–0.99; *P*=0.043). The FFR of the CCRT+AC group was significantly higher than that of the RT group (*P*<0.05). Most of the late toxicities were similar (33% *vs.* 26%, respectively; *P*=0.089). The incidence of cranial neuropathy, peripheral neuropathy, and ear injury in the CCRT+AC group was significantly higher than that in the RT group (*P*<0.05) (18).

In summary, even in NPC endemic areas, the “three cycles of CCRT plus three cycles of AC” treatment regimen can significantly improve the DFS rate, DMFS rate, and OS rate of NPC patients without increasing long-term side effects. However, whether the survival benefit for patients comes from the combined effect of concurrent and AC, or only from CCT or AC, remains to be determined.

A phase III, randomized clinical trial, also from an NPC endemic area, included 284 patients (III to IV, M_0_, AJCC 1992 staging system). A total of 141 patients were included in the CCRT treatment group (trial group) and 143 in the RT treatment (control) group. CCT was administered as a mixture of 20 mg/m^2^/d cisplatin and 400 mg/m^2^/d fluorouracil for 96 h in weeks 1 and 5. The results showed that, after a median follow-up of 65 months, the tumor recurrence rate of the CCRT and RT groups was 26.2% (37/141) and 46.2% (66/143); the five-year OS rate was 72.3% and 54.2% (*P*=0.0022); and the five-year PFS rate was 71.6% and 53% (*P*=0.0012), respectively (19). This confirmed that, compared with RT treatment, CCRT can significantly improve the survival benefits for NPC patients. A phase III, randomized clinical trial conducted by Chan et al. included 350 patients (stage N_1–3_ with at least 4-cm lymph node size in the UICC 1997 staging system), including 141 that received CCRT (trial group) and 143 that received RT (control group). CCT was performed with 40 mg/m^2^/week cisplatin. Analysis of the results showed that the five-year OS rate of patients in the control group was 58.6% (95% CI=50.9–66.2), while that of patients in the test group was 70.3% (95% CI=63.4–77.3). Cox regression analysis showed that the difference in the OS rate was statistically significant after adjusting for T stage, age, and overall stage, and the OS rate of the CCRT group was more significant than that of the RT group (*P*=0.049, HR=0.71 [95% CI=0.5–1.0]). Subgroup analysis showed that there was no difference in the OS rate for T1/T2 stage patients (*P*=0.74, HR=0.93 [95% CI=0.59–1.4]), whereas a difference was identified in the OS rate for T3/T4 stage patients (*P*=0.013, HR=0.51 [95% CI=0.3–0.88]) (20). The authors concluded that weekly CCT is a promising standard treatment strategy for patients with locally advanced NPC. The results of several meta-analyses have also shown that CCRT can significantly improve the survival benefits for patients with NPC (21–23).

The efficacy of CCRT is widely recognized. Whether AC can also bring survival benefits to patients is a further issue that we need to discuss.

## Radiotherapy Plus Adjuvant Chemotherapy Versus Radiotherapy Alone

To date, three randomized controlled trials (RCTs) have compared the efficacy of RT combined with AC and RT alone in the treatment of locally advanced NPC (**Table 2**). A trial conducted by Rossi et al. included 229 patients with locally advanced NPC. These patients achieved complete remission (CR) after the completion of RT. Subsequently, the patients were divided into two groups, one receiving RT treatment alone (*n*=116) and the other RT treatment combined with AC (*n*=113). The chemotherapy regimen was six cycles of combined vincristine, cyclophosphamide, and adriamycin (VCA). No significant difference was seen in the four-year relapse-free survival (RFS) rate (55.8% *vs.* 57.7%, respectively; *P*=0.45) and four-year OS rate (67.3% *vs.* 58.5%, respectively; *P*=0.13) between the two groups, and the mode of recurrence was also similar. Approximately 50% of the patients had treatment failure due to distant metastasis. The authors concluded that this study did not identify any additional benefit for the application of VCA chemotherapy after effective RT because the incidence of local and distant failure after RT was still high. Systemic chemotherapy should be further explored (24).

**Table 2:** Radiotherapy plus adjuvant chemotherapy VS radiotherapy alone.

References	Inclusion period	Comparison	Number of patients	Stage	Concurrent chemotherapy	AC regimen	Survival endpoints	With AC	No AC	*P*
Rossi et al. (24)	1979–1983	RT+AC/RT alone	113/116	A modified TNM system derived from the classifications of Ho’ 3 and the UICC	\	vincristine, cyclophosphamide, adriamycin	4-year OS	58.50%	67.30%	0.13
							4-year RFS	57.70%	55.80%	0.45
Chi et al. (25)	1994–1999	RT+AC/RT alone	77/77	Stage IV (AJCC/UICC 1992) disease (including T_4_N_0–1_M_0_ and TN_2–3_M_0_)	\	cisplatin, fluorouracil, leucovorin	5-year OS	54.50%	60.50%	0.5
							5-year RFS	54.40%	49.50%	0.38
Kwong et al. (26)	1995–2001	CCRT+AC/CCRT/RT+AC/RT alone	57/53/54/55	Ho’s stage T_3_ or N_2_/N_3_ or 4cm neck node, M_0_	uracil, tegafur	cisplatin, fluorouracil, vincristine, bleomycin, methotrexate	3-year OS	80.4%	83.1%	0.69
							3-year FFS	62.5%	65%	0.83
Kwong et al. (27)	1995–2001	CCRT+AC/CCRT/RT+AC/RT alone	57/53/54/55	Ho’s stage T_3_ or N_2_/N_3_ or 4cm neck node, M_0_	uracil, tegafur	cisplatin, fluorouracil, vincristine, bleomycin, methotrexate	5-year DMFS	75.1%	79.8%	0.26
							5-year FFS	60.8%	61.3%	0.99

AC, adjuvant chemotherapy; RT+AC, radiotherapy plus adjuvant chemotherapy; RT, radiotherapy; OS, overall survival; RFS, relapse-free survival; FFS, failure-free survival; DMFS, distant metastasis-free survival; T, tumor; N, node; M, metastasis; CCRT, concurrent chemoradiotherapy; CCRT+AC, concurrent chemoradiotherapy plus adjuvant chemotherapy; AJCC/UICC, American Joint Committee on Cancer and Union for International Cancer Control.

Another trial conducted in Taiwan enrolled a total of 144 NPC patients, 77 of which received RT alone, and 77 of which received nine cycles of AC (stage IV, M_0_, AJCC/UICC 1992). The AC regimen was 20 mg/m^2^ cisplatin; 2,200 mg/m^2^ 5-fluorouracil; and 120 mg/m^2^ leucine. The five-year OS and RFS of these two groups was 60.5% *vs.* 54.5% (*P*=0.50) and 49.5% *vs.* 54.4% (*P*=0.38), respectively. Cox regression analysis showed that the risk ratio of combination AC therapy to RT alone was 0.673 (*P*=0.232; 95% CI=0.352–1.288). The authors concluded that AC after RT does not improve OS or RFS compared with RT alone for patients with locally advanced NPC (25).

In 2004, Kwong et al. conducted a study with a total of 219 patients that received either CCRT or RT. Additionally, 101 patients received AC while 108 did not. The results showed that the three-year OS rate of the patients in the CCRT/RT+AC group and CCRT/RT group was 80.4% and 83.1% (*P*=0.69), while the FFS rate was 62.5% and 65% (*P*=0.83), respectively. There was no significant difference in the DMFS and LRFS rates between the two groups (*P*=0.34 and 0.15, respectively). However, multivariate regression analysis showed that CCRT was a good prognostic index (HR=0.42; *P*=0.009) for OS (26). After a median follow-up of 4.9 years, the five-year FFR of these two groups was 60.8% and 61.3%, respectively (*P*=0.99) (27).

The results of these three trials suggested that AC may not be necessary after radical RT as AC did not elicit a survival benefit.

## Concurrent Chemoradiotherapy Plus Adjuvant Chemotherapy Versus Concurrent Chemoradiotherapy

The current NCCN guidelines suggest the use of CCRT followed or not by AC as the standard treatment for locally advanced NPC (category 2 evidence). Several RCTs have been carried out to evaluate this (**Table 3**), the largest of which was trial number NCT00677118, registered on ClinicalTrials.gov. A total of 251 patients (stage III or IVA, except T_3–4_N_0_, sixth edition AJCC/UICC NPC staging criteria) treated with CCRT+AC were enrolled in the trial group, and 257 patients treated with CCRT alone were enrolled in the control group. Both groups received cisplatin CCT (40 mg/m^2^, once a week, for seven weeks). The AC was a four-week regimen of cisplatin (80 mg/m^2^) combined with 5-fluorouracil (800 mg/m^2^/d, maintained for 120 h), with a total of three cycles. The results showed that the two-year FFR of the trial group and control group was 86% and 84%, respectively (HR=0.74; 95% CI=0.49–1.10; *P*=0.13). AC combination treatment did not show any effect (28). After a median follow-up of 68.4 months, the long-term results of the trial showed that the five-year FFS rate of the trial group and control group was 75% and 71%, respectively (HR=0.88; 95% CI=0.64–1.12; *P*=0.45) (29). Combination treatment with AC still failed to improve the survival rate. As a result of this trial, the NCCN guidelines revised the recommendation of CCRT plus AC from category 1 to category 2A.

**Table 3:** Concurrent chemoradiotherapy plus adjuvant chemotherapy versus concurrent chemoradiotherapy.

References	Inclusion period	Comparison	Number of patients	Stage	Concurrent chemotherapy	AC regimen	Survival endpoints	With AC	No AC	*P*
Chen et al. (28)	2006–2010	CCRT+AC/CCRT alone	251/257	III-IV (M_0_) (except T_3-4_N_0_), 6th AJCC	cisplatin	cisplatin, fluorouracil	2-year OS	94%	92%	0.32
							2-year FFS	86%	84%	0.13
Chen et al. (29)	2006–2010	CCRT+AC/CCRT alone	251/257	III-IV (M_0_) (except T_3-4_N_0_), 6th AJCC	cisplatin	cisplatin, fluorouracil	5-year OS	83%	80%	0.35
							5-year FFS	75%	71%	0.45
Dong et al. (30)	2008–2010	CCRT+AC/CCRT alone	244/244	III and IV_A-B_, 2009 TNM	cisplatin, fluorouracil	cisplatin, fluorouracil	4-year OS	74%	72%	0.474
							4-year PFS	62%	61%	0.489
Yang et al. (31)	2006–2011	CCRT+AC/CCRT alone	76/79	III–IV_B_, 7th AJCC	cisplatin	cisplatin or nedaplatin, fluorouracil	3-year OS	73.7%	71.6%	0.44
							3-year FFS	66.9%	57.5%	0.19
Zhang et al. (32)	2003–2007	CCRT+AC/CCRT alone	93/96	II–IV_B_, AJCC	cisplatin	cisplatin, fluorouracil	5-year OS	97.83%	97.92%	0.643
							5-year PFS	71.4%	66.7%	0.96

AC, adjuvant chemotherapy; CCRT+AC, concurrent chemoradiotherapy plus adjuvant chemotherapy; OS, overall survival; PFS, progression-free survival; FFS, failure-free survival; T, tumor; N, node; M, metastasis; CCRT, concurrent chemoradiotherapy; AJCC, American Joint Committee on Cancer.

A multicenter pairing study by Dong et al. included 488 patients (stage III and IVA–B, according to the 2009 TNM classification). A total of 244 patients were enrolled in both the CCRT group and the CCRT+AC group. The CCRT regimen was PF (cisplatin+5-fluorouracil) or TP (paclitaxel+cisplatin). The PF regimen comprised the AC. The four-year OS rate, PFS rate, DMFS rate, and LRFS rate in these two groups was, respectively, 72% *vs.* 74% (HR=0.89; 95% CI=0.64–1.23; *P*=0.474), 61% *vs.* 62% (HR=0.91, 95% CI=0.68–1.20, *P*=0.489), 71% *vs.* 73% (HR=0.84, 95% CI=0.59–1.18, *P*=0.316), and 81% *vs.* 84% (HR=0.84, 95% CI=0.52–1.24, *P*=0.323). Overall, the incidence of grade 3–4 toxicity was higher in the CCRT+AC group. The authors concluded that the addition of AC after CCRT increases toxicity and cannot improve the survival of patients with locally advanced NPC (30). Several retrospective studies have also shown that the addition of AC based on CCRT did not significantly improve clinical efficacy or elicit survival benefits (31, 32).

Liang et al. (33) published a meta-analysis that included 793 patients with locally advanced NPC in five RCTs. The risk ratios for three-year OS, five-year FFS, five-year LRFS, and five-year DMFS were 1.02 (95% CI=0.89–1.15), 0.93 (95% CI=0.72–1.21), 1.07 (95% CI=0.87–1.32), and 0.95 (95% CI=0.80–1.13), respectively. There was no treatment-related death in any of the five studies. The most significant hematological and gastrointestinal toxicities were observed during the AC. Consequently, the authors concluded that, compared with CCRT alone, CCRT plus AC could not improve patient prognosis, and more toxic reactions were found during AC (33).

In 2013, OuYang et al. performed a meta-analysis of 5 RCTs that included a total of 1,187 patients with NPC. They found that patients who received additional AC treatment had a lower local recurrence rate (*P*=0.03; HR=0.71, 95% CI=0.53–0.96). However, AC had no benefit for the DMFS rate or OS rate (34).

A meta-analysis of 8 studies (a total of 2,144 patients) in 2014 showed that CCRT+AC and CCRT were significantly better than RT alone for all the parameters analyzed, but there was no significant difference between them. Although CCRT+AC was superior to CCRT alone for OS, LRFS, and DMFS, the differences were not significant (OS: HR=0.86, 95% CI=0.60–1.16; LRFS: HR=0.72, 95% CI=0.43–1.15; DMFS: HR=0.86, 95% CI=0.62–1.16). The authors could not definitely conclude that AC increases toxicity in patients with locally advanced NPC. In addition, some patients in certain states may benefit from AC, which also merits further investigation (35).

In 2015, a meta-analysis conducted by Blanchard et al. included 19 trials and collected information for a total of 4,806 NPC patients with a median follow-up period of 7.7 years. The analysis indicated that patients receiving the CCRT+AC regimen achieved the greatest benefit. The survival rates were not significantly different between the CCRT and the CCRT+AC groups; however, differences were found in baseline data between CCRT- and CCRT+AC-related clinical trials, which prevented an unbiased comparison between the two treatments (23).

Although numerous clinical trials, retrospective studies, and meta-analysis have shown that patients with NPC cannot gain significant survival benefits by continuing to receive AC after CCRT, several studies have also demonstrated that AC can provide survival benefits for patients.

A meta-analysis of 20 studies (a total of 5,144 patients) published by Ribassin-Majed et al. (2016) showed that, under the three treatment modes of CCRT+AC, CCRT, and IC+CCRT, the highest OS rate was CCRT+AC. The HRs were 0.65 (0.56–0.75), 0.77 (0.64–0.92), and 0.81 (0.63–1.04), respectively. Compared with CCRT treatment, the HRs for OS, PFS, LC, and DC in the CCRT+AC group were 0.85 (0.68–1.05), 081 (0.66–0.98), 0.70 (0.48–1.02), and 0.87 (0.61–1.25), respectively. For patient survival benefits, IC+CCRT ranked second in PFS and first in DC, while CCRT never ranked first. Compared with IC+CCRT treatment, the HRs for OS, PFS, LC, and DC in the CCRT treatment group were 0.95 (0.72–1.25), 1.13 (0.88–1.46), 1.05 (0.70–1.59), and 1.55 (0.94–2.56), respectively. The greater the number of cycles of chemotherapy, the greater the risk of acute toxicity. Therefore, the authors concluded that, compared with all the other RT/chemotherapy combinations, CCRT+AC produced the highest survival benefit. IC combined with CCRT had the greatest effect on the control of distant metastasis (36).

Although most studies have shown that AC for NPC elicits no significant survival benefit, a few studies have nonetheless concluded that AC is effective at treating NPC. Whether this is because AC can only have a significant effect on patients at certain stages or in certain states remains unknown.

## Induction Chemotherapy Plus Concurrent Chemoradiotherapy Versus Concurrent Chemoradiotherapy Plus Adjuvant Chemotherapy

In 2015, Anne W.M. Lee et al. published preliminary results of trial NPC-0501, which randomly assigned 706 patients to six treatment groups. The median duration of follow-up was 3.3 years. The results showed that there was no significant difference in the 3-year survival rate between the IC (PF regimen) + CCRT group and CCRT + AC (PF regimen) group when the analyses were adjusted for other significant factors and fractionation. Compared with the CCRT + AC (PF regimen) group, the IC (cisplatin and capecitabine [PX regimen]) + CCRT group had achieved a significant reduction in the hazards of progression (HR, 0.54; 95% CI, 0.36–0.80; *P*=0.002) and death (HR, 0.42; 95% CI, 0.25–0.70; *P*=0.001). When the PF and PX induction regimens were combined for evaluation of the IC + CCRT group versus the CCRT + AC group, unadjusted comparisons did not reach statistical significance, but adjusted comparisons indicated a reduction in the hazards of disease progression (HR, 0.67; 95% CI, 0.48–0.93; *P*=0.016) and death (HR, 0.57; 95% CI, 0.39–0.86; *P*=0.006) (37).

In 2020, Anne W.M. Lee et al. updated the results of trial NPC-0501.The median duration of follow-up was 8.4 years. In the conventional-fractionation group, the 5-year PFS of the IC + CCRT group and CCRT + AC group were (78% vs 62%; *P*=0.015), respectively. Comparison of the IC (PX regimen) + CCRT group versus CCRT + AC (PF regimen) group demonstrated better PFS (78% vs 62%; *P*=0.027) without an increase in overall late toxicity. The NPC-0501 trial is the only randomized trial to date to evaluate the survival rates of IC + CCRT versus CCRT + AC. Current study data tend to suggest that IC+CCRT is more beneficial to patients because it can significantly improve PFS and slightly improve OS without affecting advanced toxicity (38).

An individual patient data network meta-analysis by C. Petit et al. in 2019 included 20 trials (5,144 patients). The results showed that both IC and AC had the highest OS benefit. The aim of this study was to compare two treatment effects measures, HR and restricted mean survival time difference (rmstD), and not to identify the best treatment as previously published with HR. Therefore, there was no comparison between IC+CCRT and CCRT+AC in this article (39).

In 2020, a propensity score-matched analysis by Si-Qi Tang et al. selected 550 patients. It indicated that the IC + CCRT group achieved higher 5-year OS (89.3% vs 85.3%, *P*=0.119), FFS (80.2% vs 79.0%, *P*=0.722) and DMFS (87.4% vs84.4%, *P*=0.322) compared with CCRT + AC, although this was statistically non-significant. Subgroup analysis revealed that CCRT + AC was associated with significantly improved LRRFS (HR=0.18, 95% CI 0.04–0.79, *P*=0.010) in the T4 subgroup (40).

So far, there have been few studies comparing IC+CCRT and CCRT+AC. According to the few studies currently available, the role of CCRT+AC is uncertain, and AC may still play a role in patients with high risk factors.

## Stratified Analysis of Various Factors

The OS rate and other survival endpoints of NPC are closely related to the clinical stage, especially the number of affected lymph nodes. Overall, the higher the N staging, the greater the possibility of distant metastasis. Additionally, the higher the staging, the shorter the survival time.

Chen et al. carried out a retrospective analysis of patients with NPC that included only stage II NPC patients (AJCC/UICC, seventh edition staging). Of the 162 patients analyzed, 80 received CCRT, 40 received CCRT+AC, and 42 received RT. All of the patients were treated with IMRT. After a median follow-up of 56 months, the three groups showed similar five-year OS rates (respectively 93.9%, 95.0%, and 95.2%; *P*=0.937), five-year LRFS rates (respectively 96.8%, 94.9%, and 93.0%; *P*=0.756), five-year DMFS rates (91.1%, 97.5%, and 100%, respectively; *P*=0.185), and five-year FFS rates (84.9%, 92.5%, and 93.0%, respectively; *P*=0.597). Univariate and multivariate analysis showed that the older the patient, the lower the LRFS and FFR rates. There were more acute toxic reactions among patients in the CCRT and CCRT+AC groups, especially myelosuppression, liver dysfunction, gastrointestinal reactions (nausea/vomiting), and weight loss. CCRT with or without AC could not improve the survival of patients with stage II NPC, and the treatment-related acute toxicity was significantly higher than that for IMRT treatment alone (41).

IMRT has good dosimetric advantages and conformability. It can not only protect the surrounding normal tissues and organs but also further improve the LC and OS rates of patients with NPC. Therefore, for early-stage NPC, radical IMRT may be sufficient to achieve the greatest therapeutic benefits.

A retrospective study by Zhong et al. in 2017 showed that there were no significant differences in the one-, two-, and three-year OS, LRFS, and DMFS rates between CCRT+AC and CCRT-only treatments. The authors subsequently performed a stratified analysis according to different T, N, and clinical stages. The results showed that in stages III, IV, and T_4_ (seventh edition AJCC/UICC NPC staging criteria), the OS, LRFS, and DMFS rates were also not significantly different. However, this study had some limitations. First, the follow-up time was very short, and only a three-year survival analysis was undertaken. Second, the sample size was small for the stratified analysis. Additionally, the number of CCT and AC cycles differed (42).

In 2014, Liang et al. reported a retrospective analysis of 260 patients with NPC (seventh edition AJCC/UICC NPC staging criteria). There were 130 patients in both the CCRT+AC group and the CCRT group. The patients were matched according to age, gender, WHO histology, T stage, N stage, and RT technique used. After a follow-up of 42.1 months, the RR for OS, LRFS, DMFS, and FFS between these two groups were 0.77 (95% CI=0.37–1.57), 1.00 (95% CI=0.37–2.71), 1.15 (95% CI=0.56–2.37), and 1.26 (95% CI=0.6–2.28), respectively. There was no significant difference in the survival rate between the two groups. A stratified analysis of tumor clinical stage indicated that the curative effect of CCRT+AC on patients with N_2–3_ disease was marginally significant (HR=0.35, 95% CI=0.11–1.06, *P*=0.052). In this study, no significant benefit in survival rate was shown after CCRT+AC treatment. However, Liang et al. observed a borderline significant difference in OS favoring CCRT+AC treatment in patients with N2–3 disease. Therefore, the authors believe that after CCRT treatment, stage N2–3 NPC might also need further treatment with AC (43).

Xu et al. undertook a retrospective study in 2011, comparing the results of different combinations of RT and chemotherapy in N_3_ stage NPC patients. All patients with NPC were staged according to the AJCC 2002 criteria. Two-dimensional RT was used. There were 15 cases in the CCRT+AC treatment group and 37 in the CCRT group. The five-year OS for the two groups was 80% and 54.2% (*P*<0.05), while the five-year DMFS was 71.1% and 51.4% (*P*<0.001), respectively. The analysis indicated that the CCRT+AC regimen was more effective at treating N_3_ stage NPC. However, this was not a RCT, and the sample size was very small (44).

In 2018, Zhang and colleagues analyzed the efficacy and safety of CCRT plus S-l AC in the treatment of stage N_3_ NPC. A total of 44 patients were enrolled and completed at least two cycles of CCT and four of AC. The total effective rate was 100.0%. The three-year OS rate was 86.4%, the DMFS rate was 84.1%, the LC rate was 97.7%, and the PFS rate was 81.8%. Although the analysis indicated that CCRT+S-1 AC provided a good survival benefit for stage N_3_ NPC patients, no group comparison was performed in this study; therefore, the conclusion that S-l AC has survival benefits remains to be further confirmed (45). Another study also suggested that AC may provide benefits for stage N_3_ NPC patients (46).

Numerous studies have shown that AC is effective at treating late-stage NPC; however, they all have shortcomings in experimental design, and large, multicenter, prospective RCTs are still needed to evaluate whether CCRT combined with AC is superior to CCRT alone in the treatment of locally advanced NPC.

Epstein–Barr virus (EBV) is a human herpesvirus with a linear, double-stranded DNA genome. More than 90% of adults have been infected with EBV, and EBV will continue to lurk in human B lymphocytes. This implies that EBV DNA in the plasma of NPC patients may originate from both NPC cells and B lymphocytes. However, increasing evidence has shown that EBV DNA in the plasma of patients with NPC comes primarily from free, short DNA fragments in NPC cells. Because of the specificity and high sensitivity of plasma EBV DNA detection, EBV DNA in tissue or plasma can also be used to judge the primary disease of cervical lymph node metastasis (47). In the clinical staging of NPC (AJCC eighth edition), if the patient has cervical lymph node metastasis and is EBV DNA-positive, even if no tumor is found in the nasopharynx, the disease can be defined as stage T0 NPC. Additionally, EBV has been suggested to be an independent risk factor affecting the prognosis of patients with NPC.

Twu et al. carried out a retrospective study that included 85 patients. One week after radical RT plus IC or CCT, EBV DNA could still be continuously detected in the plasma. Among the 85 patients, 33 received AC (two capsules of tegafur–uracil, twice a day) for one year. The other 52 patients did not receive any AC. There was no significant difference in age, gender, pathological type, manifestation, T type, N type, or overall stage between the two groups. After a median follow-up of 70 months, the recurrence rates of tumors in patients receiving or not receiving AC were 45.5% and 71.2%, respectively (*P*=0.0323). There was a significant difference in the rate of distant metastasis between the two groups (*P*=0.034), but no difference in local recurrence rates. The five-year OS rate of the two groups was 71.6% and 28.7%, respectively. AC significantly improved the OS rate (HR=0.27, 95% CI=0.17–0.55, *P*<0.0001). The limitation of this study was that it was not a randomized controlled clinical trial. Moreover, the sample size was too small, and the treatment plan was not uniform (48).

In July 2018, Chan et al. published the results of the Hong Kong Nasopharyngeal Cancer Research Group Trial No. 0502. In this prospective, randomized, controlled clinical trial, 789 patients with NPC (stage IIB–IVB) who had completed radical RT or RT combined with chemotherapy (staged according to the sixth edition of AJCC/UICC) were screened, 104 of which were positive for plasma EBV DNA after RT. They were randomly assigned to an AC group and a clinical observation group. The AC regimen was gemcitabine 1000 mg/m^2^ on d1 and 8, plus cisplatin 40 mg/m^2^, for a total of 6 cycles. After a median follow-up of 6.6 years, there was no statistical difference in survival rates between the AC group and the clinical observation group. The five-year OS rate was 64.0% *vs.* 67.8%, *P*=0.79); the DFS rate was 49.3% *vs.* 54.7%, *P*=0.75; the DMFS rate was 58.9% *vs.* 63.8%, *P*=0.84; and the LRFS rate was 54.6% *vs.* 59.1%, *P*=0.68, respectively (43). The results of this study suggested that increasing the AC dosage does not benefit the survival of such high-risk patients. The authors concluded that the resistance of potential subclinical lesions to platinum may be one of the reasons for the negative results after concurrent cisplatin chemotherapy. This RCT was the first of its kind to be published and was an important step toward individualized NPC treatment. Although the results were negative, it nevertheless has important scientific significance (49).

The current trend for tumor treatment involves individualized therapy. Consequently, risk stratification of patients with NPC according to specific survival and prognostic factors is one of the areas of research focus.

In 2015, Liu et al. performed a retrospective analysis of a cohort of 400 patients with high-risk NPC. The definition of high-risk NPC included (1) cervical lymph node >6 cm; (2) supraclavicular lymph node metastasis; (3) skull base destruction/intracranial invasion + multiple lymph node metastasis; or (4) multiple cervical lymph node metastasis, with the largest lymph node measuring >4 cm. All the patients completed a full course of CCRT or IC+RT. Of the 400 patients, 154 received AC after RT (two capsules of tegafur–uracil, twice a day) for one year, while the other 249 received only RT. After a median follow-up of 72 months, the tumor recurrence rate in the AC+RT group and that in the RT-only group was 31.8% (49/154) and 42.2% (105/249), respectively; the five-year DMFS rate was 82.1% and 68.5% (*P*=0.0018) and the LRFS rate was 84.3% and 82.6% (*P*=0.7848), respectively. The OS rate of patients who received AC was significantly higher than that of patients who received only RT (80.5% *vs.* 66.3%, respectively; *P*=0.0001) (50).

In 2017, Liang et al. reviewed the clinical data of 511 patients with NPC who had received or not AC after CCRT. In total, 177 patients received CCRT and 334 received CCRT+AC. Survival analysis showed that >45 years old, serum albumin levels ≤42 g/L, T_3–4_ stage, and N_2–3_ stage were important independent prognostic factors for OS. Using these four risk factors, the authors established a prognostic model for OS, with patients presenting with 0–1 risk factor being considered low-risk and those with 2–4 risk factors being considered high-risk. The results showed that the five-year OS of patients in the high-risk group was significantly improved (HR=0.61, 95% CI=0.30~0.96, *P*=0.03) after the addition of AC to the CCRT, whereas there was no survival benefit for patients in the low-risk group (51).

Because of the large sample size, unified chemotherapy regimen, RT technique used, and long follow-up time, the results of the two above-mentioned retrospective studies strongly suggest that AC can improve the survival of high-risk NPC patients.

In clinical trials, the proportion of patients receiving enough cycles of AC is generally low owing to the economic status of patients, compliance, as well as other reasons, all of which may affect the reported efficacy of AC. Whether the effect of AC is related to the number of cycles also merits further discussion.

In a retrospective analysis, Li et al. (2007) analyzed 253 patients with NPC (according to Fuzhou [1992] stage, T_1–4_N_0–3_M_0_, stages III and IV). The patients were divided into 4 groups according to the different modes of treatment: a RT-only group (group 0, *n*=69); a two-cycle CCRT group (group 2, *n*=67); a two-cycle CCRT + one-cycle AC group (group 2 + 1, *n*=47); and a two-cycle CCRT + two-cycle AC group (group 2 + 2, *n*=70). Survival analysis showed that the five-year OS rate (*P*=0.988), DFS rate (*P*=0.724), LRFS rate (*P*=0.257), and DMFS rate (*P*=0.315) were similar among groups 2, 2 + 1, and 2 + 2 (52).

In 2013, Lin et al. reviewed the data for 181 patients with locally advanced NPC who were treated with CCRT and AC. After a median follow-up of 40 months, the five-year OS rate for patients treated with and without AC was 83.6% and 66.7%, respectively (*P*=0.027). The prognosis of patients who received 2–3 cycles of AC was better than that of patients who did not receive AC or those who received one cycle of AC (53).

## Conclusions

In summary, compared with RT alone, AC after CCRT is significantly beneficial for the survival of patients with locally advanced NPC, but no matter in the comparison between RT+AC and RT alone, or the comparison between CCRT+AC and CCRT. AC has not been shown to be effective in most clinical studies, whereas several clinical trials have confirmed the effectiveness of CCRT. This suggests that the survival benefit associated with the “CCRT plus AC” treatment mode is likely to be derived from CCT rather than AC. NCCN guidelines recommend CCRT+AC as category 2A. At present, relatively few studies have analyzed the significance of AC, especially in multi-factor stratified analysis. AC remains important for the treatment of high-risk NPC. For patients with locally advanced NPC who cannot tolerate sufficient doses of CCT after receiving IC, a prospective phase II clinical study showed that the combination of IC and AC elicited five-year OS, LC, and DC rates of 82.1%, 92.2%, and 89.0%, respectively. This combination provides a good treatment choice for patients with locally advanced NPC who cannot tolerate CCRT (54). The role of AC in treating locally advanced NPC, based on the studies presented, remains undefined but is associated with increased toxicity. For accurate medical treatment, it is necessary to unify the high-risk factors for NPC, identify more accurate prognostic indicators, determine the appropriate number of AC cycles, explore new chemotherapeutic regimens, and select people who are suitable for AC. Additional rigorous prospective clinical trials are required to provide more accurate treatment strsategies for patients with locally advanced NPC.

## Author Contributions

LiS wrote the first draft of this article. LeS contributed to manuscript revision. LFS approved the submitted version. All authors contributed to the article and approved the submitted version.

## Conflict of Interest

The authors declare that the research was conducted in the absence of any commercial or financial relationships that could be construed as a potential conflict of interest.

## References

[1] Cao SM Simons MJ Qian CN. The prevalence and prevention of nasopharyngeal carcinoma in China. Chin J Cancer (2011) 30:114–9. 10.5732/cjc.010.10377 PMC4013340 21272443

[2] Bray F Ferlay J Soerjomataram I Siegel RL Torre LA Jemal A. Global cancer statistics 2018: GLOBOCAN estimates of incidence and mortality worldwide for 36 cancers in 185 countries. CA Cancer J Clin (2018) 68:394–424. 10.3322/caac.21492 30207593

[3] Ferlay J Colombet M Soerjomataram I Mathers C Parkin DM Pineros M. Estimating the global cancer incidence and mortality in 2018: GLOBOCAN sources and methods. Int J Cancer (2019) 144:1941–53. 10.1002/ijc.31937 30350310

[4] Chen Y-P Chan ATC Le Q-T Blanchard P Sun Y Ma J. Nasopharyngeal carcinoma. Lancet (2019) 394:64–80. 10.1016/s0140-6736(19)30956-0 31178151

[5] Wei WI Sham JST. Nasopharyngeal carcinoma. Lancet (2005) 365:2041–54. 10.1016/s0140-6736(05)66698-6 15950718

[6] Mao YP Xie FY Liu LZ Sun Y Li L Tang LL. Re-evaluation of 6th edition of AJCC staging system for nasopharyngeal carcinoma and proposed improvement based on magnetic resonance imaging. Int J Radiat Oncol Biol Phys (2009) 73:1326–34. 10.1016/j.ijrobp.2008.07.062 19153016

[7] Lin S Lu JJ Han L Chen Q Pan J. Sequential chemotherapy and intensity-modulated radiation therapy in the management of locoregionally advanced nasopharyngeal carcinoma: experience of 370 consecutive cases. BMC Cancer (2010) 10:39. 10.1186/1471-2407-10-39 20146823 PMC2836300

[8] Su SF Han F Zhao C Huang Y Chen CY Xiao WW. Treatment outcomes for different subgroups of nasopharyngeal carcinoma patients treated with intensity-modulated radiation therapy. Chin J Cancer (2011) 30:565–73. 10.5732/cjc.010.10547 PMC4013407 21801605

[9] Wong FC Ng AW Lee VH Lui CM Yuen KK Sze WK. Whole-field simultaneous integrated-boost intensity-modulated radiotherapy for patients with nasopharyngeal carcinoma. Int J Radiat Oncol Biol Phys (2010) 76:138–45. 10.1016/j.ijrobp.2009.01.084 19646824

[10] Lee AW Lin JC Ng WT. Current management of nasopharyngeal cancer. Semin Radiat Oncol (2012) 22:233–44. 10.1016/j.semradonc.2012.03.008 22687948

[11] Al-Sarraf M LeBlanc M Giri PG Fu KK Cooper J Vuong T. Chemoradiotherapy versus radiotherapy in patients with advanced nasopharyngeal cancer: phase III randomized Intergroup study 0099. J Clin Oncol (1998) 16:1310–7. 10.1200/JCO.1998.16.4.1310 9552031

[12] Al-Sarraf M Reddy M. Nasopharyngeal carcinoma. Curr Treat Options Oncol (2002) 3:21–32. 10.1007/s11864-002-0038-8 12057084

[13] Wee J Tan EH Tai BC Wong HB Leong SS Tan T. Randomized trial of radiotherapy versus concurrent chemoradiotherapy followed by adjuvant chemotherapy in patients with American Joint Committee on Cancer/International Union against cancer stage III and IV nasopharyngeal cancer of the endemic variety. J Clin Oncol (2005) 23:6730–8. 10.1200/JCO.2005.16.790 16170180

[14] Lee AW Lau WH Tung SY Chua DT Chappell R Xu L. Preliminary results of a randomized study on therapeutic gain by concurrent chemotherapy for regionally-advanced nasopharyngeal carcinoma: NPC-9901 Trial by the Hong Kong Nasopharyngeal Cancer Study Group. J Clin Oncol (2005) 23:6966–75. 10.1200/JCO.2004.00.7542 16192584

[15] Lee AWM Tung SY Ng WT Lee V Ngan RKC Choi HCW. A multicenter, phase 3, randomized trial of concurrent chemoradiotherapy plus adjuvant chemotherapy versus radiotherapy alone in patients with regionally advanced nasopharyngeal carcinoma: 10-year outcomes for efficacy and toxicity. Cancer (2017) 123:4147–57. 10.1002/cncr.30850 28662313

[16] Ng WT Tung SY Lee V Ngan RKC Choi HCW Chan LLK. Concurrent-Adjuvant Chemoradiation Therapy for Stage III-IVB Nasopharyngeal Carcinoma-Exploration for Achieving Optimal 10-Year Therapeutic Ratio. Int J Radiat Oncol Biol Phys (2018) 101:1078–86. 10.1016/j.ijrobp.2018.04.069 29885997

[17] Chen Y Liang SB Mao YP Zong JF Tang LL Guo Y. A Prospective Randomized Trial of Concurrent Chemoradiotherapy plus Adjuvant Chemotherapy in Patients with Locoregionally Advanced Nasopharyngeal Carcinoma. Chin J Clin Oncol (2008) 35:785–9. 10.3969/j.issn.1000-8179.2008.14.002 18472356

[18] Chen Y Sun Y Liang SB Zong JF Li WF Chen M. Progress report of a randomized trial comparing long-term survival and late toxicity of concurrent chemoradiotherapy with adjuvant chemotherapy versus radiotherapy alone in patients with stage III to IVB nasopharyngeal carcinoma from endemic regions of China. Cancer (2013) 119:2230–8. 10.1002/cncr.28049 23576020

[19] Lin JC Jan JS Hsu CY Liang WM Jiang RS Wang WY. Phase III study of concurrent chemoradiotherapy versus radiotherapy alone for advanced nasopharyngeal carcinoma: positive effect on overall and progression-free survival. J Clin Oncol (2003) 21:631–7. 10.1200/JCO.2003.06.158 12586799

[20] Chan AT Leung SF Ngan RK Teo PM Lau WH Kwan WH. Overall survival after concurrent cisplatin-radiotherapy compared with radiotherapy alone in locoregionally advanced nasopharyngeal carcinoma. J Natl Cancer Inst (2005) 97:536–9. 10.1093/jnci/dji084 15812080

[21] Zhang L Zhao C Ghimire B Hong MH Liu Q Zhang Y. The role of concurrent chemoradiotherapy in the treatment of locoregionally advanced nasopharyngeal carcinoma among endemic population: a meta-analysis of the phase iii randomized trials. BMC Cancer (2010) 10:558. 10.1186/1471-2407-10-558 20950416 PMC2970609

[22] Langendijk JA Leemans CR Buter J Berkhof J Slotman BJ. The additional value of chemotherapy to radiotherapy in locally advanced nasopharyngeal carcinoma: a meta-analysis of the published literature. J Clin Oncol (2004) 22:4604–12. 10.1200/JCO.2004.10.074 15542811

[23] Blanchard P Lee A Marguet S Leclercq J Ng WT Ma J. Chemotherapy and radiotherapy in nasopharyngeal carcinoma: an update of the MAC-NPC meta-analysis. Lancet Oncol (2015) 16:645–55. 10.1016/s1470-2045(15)70126-9 25957714

[24] Rossi A Molinari R Boracchi P Del Vecchio M Marubini E Nava M. Adjuvant chemotherapy with vincristine, cyclophosphamide, and doxorubicin after radiotherapy in local-regional nasopharyngeal cancer: results of a 4-year multicenter randomized study. J Clin Oncol (1988) 6:1401–10. 10.1200/JCO.1988.6.9.1401 3047335

[25] Chi KH Chang YC Guo WY Leung MJ Shiau CY Chen SY. A phase III study of adjuvant chemotherapy in advanced nasopharyngeal carcinoma patients. Int J Radiat Oncol Biol Phys (2002) 52:1238–44. 10.1016/s0360-3016(01)02781-x 11955734

[26] Kwong DL Sham JS Au GK Chua DT Kwong PW Cheng AC. Concurrent and adjuvant chemotherapy for nasopharyngeal carcinoma: a factorial study. J Clin Oncol (2004) 22:2643–53. 10.1200/JCO.2004.05.173 15226332

[27] Kwong DLW Sham JST Au GKH. Five-year Update on a Randomized Factorial Study on Concurrent and Adjuvant Chemotherapy for Advanced Nasopharyngeal Carcinoma. Int J Radiat Oncol Biol Phys (2006) 66:S15–S6. 10.1016/j.ijrobp.2006.07.1340

[28] Chen L Hu CS Chen XZ Hu GQ Cheng ZB Sun Y. Concurrent chemoradiotherapy plus adjuvant chemotherapy versus concurrent chemoradiotherapy alone in patients with locoregionally advanced nasopharyngeal carcinoma: a phase 3 multicentre randomised controlled trial. Lancet Oncol (2012) 13:163–71. 10.1016/s1470-2045(11)70320-5 22154591

[29] Chen L Hu CS Chen XZ Hu GQ Cheng ZB Sun Y. Adjuvant chemotherapy in patients with locoregionally advanced nasopharyngeal carcinoma: Long-term results of a phase 3 multicentre randomised controlled trial. Eur J Cancer (2017) 75:150–8. 10.1016/j.ejca.2017.01.002 28235726

[30] Dong YY Xiang C Lu JX Su YX Pan YF Cai R. Concurrent chemoradiotherapy plus adjuvant chemotherapy versus concurrent chemoradiotherapy in locoregionally advanced nasopharyngeal carcinoma: a matched-pair multicenter analysis of outcomes. Strahlenther Onkol (2016) 192:394–402. 10.1007/s00066-016-0970-3 27215563

[31] Yang S Lin S Fu Q Cai B Kong F Huang G. The effect of adjuvant chemotherapy on survival in patients with residual nasopharyngeal carcinoma after undergoing concurrent chemoradiotherapy. PLoS One (2015) 10:e0120019. 10.1371/journal.pone.0120019 25799566 PMC4370638

[32] Zhang W Dou H Lam C Liu J Zhou J Liu Y. Concurrent chemoradiotherapy with or without adjuvant chemotherapy in intermediate and locoregionally advanced nasopharyngeal carcinoma. Tumour Biol (2013) 34:1729–36. 10.1007/s13277-013-0710-6 23436047

[33] Liang ZG Zhu XD Zhou ZR Qu S Du YQ Jiang YM. Comparison of concurrent chemoradiotherapy followed by adjuvant chemotherapy versus concurrent chemoradiotherapy alone in locoregionally advanced nasopharyngeal carcinoma: a meta-analysis of 793 patients from 5 randomized controlled trials. Asian Pac J Cancer Prev (2012) 13:5747–52. 10.7314/apjcp.2012.13.11.5747 23317250

[34] OuYang PY Xie C Mao YP Zhang Y Liang XX Su Z. Significant efficacies of neoadjuvant and adjuvant chemotherapy for nasopharyngeal carcinoma by meta-analysis of published literature-based randomized, controlled trials. Ann Oncol (2013) 24:2136–46. 10.1093/annonc/mdt146 23613477

[35] Chen YP Wang ZX Chen L Liu X Tang LL Mao YP. A Bayesian network meta-analysis comparing concurrent chemoradiotherapy followed by adjuvant chemotherapy, concurrent chemoradiotherapy alone and radiotherapy alone in patients with locoregionally advanced nasopharyngeal carcinoma. Ann Oncol (2015) 26:205–11. 10.1093/annonc/mdu507 25355717

[36] Ribassin-Majed L Marguet S Lee AWM Ng WT Ma J Chan ATC. What Is the Best Treatment of Locally Advanced Nasopharyngeal Carcinoma? An Individual Patient Data Network Meta-Analysis. J Clin Oncol (2017) 35:498–505. 10.1200/JCO.2016.67.4119 27918720 PMC5791836

[37] Lee A Ngan R Tung S Cheng A Kwong D Lu T. Preliminary results of trial NPC-0501 evaluating the therapeutic gain by changing from concurrent-adjuvant to induction-concurrent chemoradiotherapy, changing from fluorouracil to capecitabine, and changing from conventional to accelerated radiotherapy fractionation in patients with locoregionally advanced nasopharyngeal carcinoma. Cancer (2015) 121:1328–38. 10.1002/cncr.29208 25529384

[38] Lee A Ngan R Ng W Tung S Cheng A Kwong D. NPC-0501 trial on the value of changing chemoradiotherapy sequence, replacing 5-fluorouracil with capecitabine, and altering fractionation for patients with advanced nasopharyngeal carcinoma. Cancer (2020) 126:3674–88. 10.1002/cncr.32972 32497261

[39] Petit C Blanchard P Pignon J Lueza B. Individual patient data network meta-analysis using either restricted mean survival time difference or hazard ratios: is there a difference? A case study on locoregionally advanced nasopharyngeal carcinomas. Syst Rev (2019) 8:96. 10.1186/s13643-019-0984-x 30987679 PMC6463649

[40] Tang S Xu C Wang X Tang L Li W Chen L. Induction versus adjuvant chemotherapy combined with concurrent chemoradiotherapy in locoregionally advanced nasopharyngeal carcinoma: A propensity score-matched analysis. Oral Oncol (2020) 105:104686. 10.1016/j.oraloncology.2020.104686 32283514

[41] Chen KH Zhu XD Li L Qu S Liang ZQ Liang X. Comparison of the efficacy between concurrent chemoradiotherapy with or without adjuvant chemotherapy and intensity-modulated radiotherapy alone for stage II nasopharyngeal carcinoma. Oncotarget (2016) 7:69041–50. 10.18632/oncotarget.11978 PMC5356610 27634892

[42] Zhong Q Zhu X Li L Qu S Liang Z Zeng F. IMRT combined with concurrent chemotherapy plus adjuvant chemotherapy versus IMRT combined with concurrent chemotherapy alone in patients with nasopharyngeal carcinoma. Oncotarget (2017) 8:39683–94. 10.18632/oncotarget.14799 PMC5503643 28147309

[43] Liang Z Zhu X Li L Qu S Liang X Liang Z. Concurrent chemoradiotherapy followed by adjuvant chemotherapy compared with concurrent chemoradiotherapy alone for the treatment of locally advanced nasopharyngeal carcinoma: a retrospective controlled study. Curr Oncol (2014) 21:e408–17. 10.3747/co.21.1777 PMC4059804 24940100

[44] Xu T Hu C Gao Y Zhang Y Wu Y He X. Treatment Outcomes of Different Chemotherapy Sequences in N3 Stage Nasopharyngeal Carcinoma. Int J Radiat Oncol Biol Phys (2010) 78:S467–S8. 10.1016/j.ijrobp.2010.07.1097

[45] Zhang S Zhou L Huang X Lin S. A retrospective study of concurrent chemoradiotherapy plus S-1 adjuvant chemotherapy on curative effect for treatment of patients with N3 stage nasopharyngeal carcinoma. Cancer Manage Res (2018) 10:1705–11. 10.2147/cmar.S165804 PMC6025766 29983590

[46] Xu T Shen C Ou X He X Ying H Hu C. The role of adjuvant chemotherapy in nasopharyngeal carcinoma with bulky neck lymph nodes in the era of IMRT. Oncotarget (2016) 7:21013–22. 10.18632/oncotarget.7849 PMC4991508 26942700

[47] Li JG Ling SJ Chen XZ. Research progress of plasma EBV DNA detection in the diagnosis and treatment of nasopharyngeal carcinoma. Chin J Clin Oncol (2018) 45:487–91. 10.3969/j.issn.1000-8179.2018.10.172.[inChinese

[48] Twu CW Wang WY Chen CC Liang KL Jiang RS Wu CT. Metronomic adjuvant chemotherapy improves treatment outcome in nasopharyngeal carcinoma patients with postradiation persistently detectable plasma Epstein-Barr virus deoxyribonucleic acid. Int J Radiat Oncol Biol Phys (2014) 89:21–9. 10.1016/j.ijrobp.2014.01.052 24725686

[49] Chan ATC Hui EP Ngan RKC Tung SY Cheng ACK Ng WT. Analysis of Plasma Epstein-Barr Virus DNA in Nasopharyngeal Cancer After Chemoradiation to Identify High-Risk Patients for Adjuvant Chemotherapy: A Randomized Controlled Trial. J Clin Oncol (2018) 36:JCO2018777847. 10.1200/JCO.2018.77.7847 29989858

[50] Liu YC Wang WY Twu CW Jiang RS Liang KL Wu CT. Prognostic impact of adjuvant chemotherapy in high-risk nasopharyngeal carcinoma patients. Oral Oncol (2017) 64:15–21. 10.1016/j.oraloncology.2016.11.008 28024719

[51] Liang ZG Chen XQ Lin GX Yu BB Chen KH Zhong QL. Significant survival benefit of adjuvant chemotherapy after concurrent chemoradiotherapy in locally advanced high-risk nasopharyngeal carcinoma. Sci Rep (2017) 7:41449. 10.1038/srep41449 28150694 PMC5288719

[52] Li DR Lin ZX Li DS Chen CZ Chen ZJ. Effect of radiotherapy combined with different cycles of chemotherapy on the survival of locally advanced nasopharyngeal carcinoma. Chin J Radiat Oncol (2008) 17:399–400. 10.3321/j.issn:1004-4221.2008.05.020

[53] Lin CC Chen TT Lin CY Hsieh CY Lin PH Chien CR. Prognostic analysis of adjuvant chemotherapy in patients with nasopharyngeal carcinoma. Future Oncol (2013) 9:1469–76. 10.2217/fon.13.100 24106898

[54] Wu M Ou D He X Hu C. Long-term results of a phase II study of gemcitabine and cisplatin chemotherapy combined with intensity-modulated radiotherapy in locoregionally advanced nasopharyngeal carcinoma. Oral Oncol (2017) 73:118–23. 10.1016/j.oraloncology.2017.08.016 28939063

